# Grain growth prediction based on data assimilation by implementing 4DVar on multi-phase-field model

**DOI:** 10.1080/14686996.2017.1378921

**Published:** 2017-10-30

**Authors:** Shin-ichi Ito, Hiromichi Nagao, Tadashi Kasuya, Junya Inoue

**Affiliations:** ^a^ Earthquake Research Institute, The University of Tokyo, Tokyo, Japan.; ^b^ Graduate School of Information Science and Technology, The University of Tokyo, Tokyo, Japan.; ^c^ Graduate School of Engineering, The University of Tokyo, Tokyo, Japan.; ^d^ Research Center for Advanced Science and Technology, The University of Tokyo, Tokyo, Japan.

**Keywords:** Grain growth, data assimilation, Bayesian statistics, prediction method, phase field model, uncertainty quantification

## Abstract

We propose a method to predict grain growth based on data assimilation by using a four-dimensional variational method (4DVar). When implemented on a multi-phase-field model, the proposed method allows us to calculate the predicted grain structures and uncertainties in them that depend on the quality and quantity of the observational data. We confirm through numerical tests involving synthetic data that the proposed method correctly reproduces the true phase-field assumed in advance. Furthermore, it successfully quantifies uncertainties in the predicted grain structures, where such uncertainty quantifications provide valuable information to optimize the experimental design.

## Graphical Abstract

**Figure:**
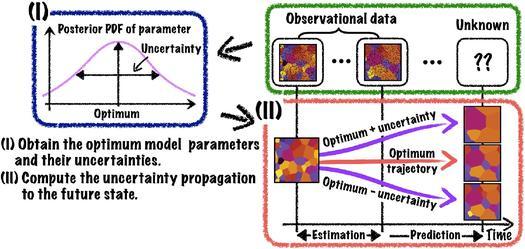

## 1. Introduction

Controlling the temporal evolution of grain structures in metals is a crucial issue in obtaining materials with desirable mechanical properties [1–3]. To control temporal evolution, predicting the dynamics of grain growth is necessary by considering the minimization of interfacial energy among grains with different crystallographic orientations. Phase-field (PF) models [4–10] have often been used to simulate such dynamics in materials science. Since PF models allow us to simulate the complex morphological dynamics of grain structures by modeling interactions among grains, simulations using these models help us understand and predict their dynamics with the goal of the efficient development of materials. However, PF models need many phenomenological parameters that are not directly observable in experiments. Thus, establishing a methodology to estimate these parameters from limited observational/experimental data is necessary to predict the dynamics of grain structures. We emphasize here that an evaluation of the uncertainties in the estimated parameters also provides valuable information, since the uncertainties affect the results of the simulated grain structures and, hence, the mechanical properties of metals.

Data assimilation (DA), which integrates simulation models and observational/experimental data on the basis of Bayesian statistics, is a computational technique to estimate unobserved states and/or parameters involved in simulation models [11]. It has been applied to many fields of science [12–19]. In general, DA can be classified into online and offline DAs. Online DA sequentially estimates states referring to continuously entered observational data, where a sequential Bayesian filter, such as a particle filter [20,21] or the ensemble Kalman filter [22,23], improves the simulation in accordance with the data. A naïve implementation of online DA is often impractical because of the massive amount of computations needed, proportional to 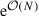, where *N*-pagination

is the dimensionality of the variables. Therefore, online DA does not seem appropriate for most PF models, where *N* easily increases when a fine grid spacing is adopted for the accurate description of the boundaries of different grains.

On the contrary, an offline DA searches the optimum initial states that best match the observed time series. The representative method for offline DA is a four-dimensional variational method (4DVar) [24,25], which is the main subject of this paper. The number of computations needed to obtain optimum initial states does not depend on *N* such that 4DVar is applicable to even massive simulation models, such as PF models. Although the conventional 4DVar has a serious disadvantage in that it only provides estimates of initial states but cannot evaluate their uncertainties, Ito et al. [19] solved this problem by establishing a new 4DVar that enables us to obtain not only the optimum, but also the associated uncertainty using a second-order adjoint (SOA) method [26–28]. This new 4DVar is appropriate for DA based on PF models because it can quantify several uncertainties of interest within a practical number of computations. The authors showed the validity of the new 4DVar by applying it to the most fundamental PF model, the Kobayashi PF model [4], which describes the growth of a single grain driven by a chemical reaction between it and its parent metal. However, the validation is inadequate from the perspective of application to more complex PF models because the dynamics of grain growth are mainly driven by interfacial energy, and the chemical reaction is a secondary factor.

In this paper, we investigate whether the new 4DVar works well in the case of a PF model that describes interface-driven dynamics. Moreover, we propose a DA procedure that allows us to investigate the extent to which uncertainties in the estimated parameters influence the prediction of the temporal evolution of grain structures. The proposed DA procedure based on the new 4DVar is expected to provide valuable information concerning the optimization of experimental design, such as regarding the number of experiments or the observation intervals. The remainder of this paper is organized as follows: Section 2 explains the experimental setup used in this paper and the PF model to describe interface-driven grain growth. Section 3 describes the procedure to predict the temporal evolution of the grain structure. Section 4 validates the proposed procedure through numerical tests and Section 5 contains a discussion of the results. Section 6 states the conclusions and directions for future work in the area.

## 2. Observational data and simulation model

### 2.1. Observational data

**Figure 1.**
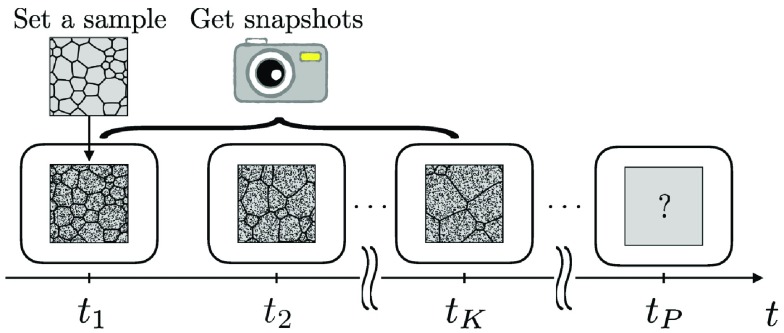
Experimental setup used in this paper. The rounded rectangles indicate a heating furnace kept in isothermal conditions. The grain structures in the furnace were observed as snapshots contaminated by noise.

When the grain structure of a metal is evolved in an experiment, a sample of the metal is placed in isothermal conditions and its grain structure is observed (see Figure 1).

In this situation, we can only observe snapshots of the grain structure rougher than those observed before placing the sample into the furnace, since the grain structures in the furnace would be contaminated by noise. We assumed that we had placed a sample in the furnace at 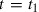, and observed its grain structures at 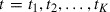. Note that the grain structure at 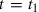 in the furnace was observed with noise, whereas the original, true grain structure was already known as it could be observed accurately before placing the sample in the furnace. For the experimental setup, the problem to be considered in this paper was estimating the grain structures at 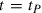 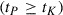 by using the observed snapshots at 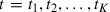. Solving the predictive problem tells us how the grains are distributed at a future time, and hence is very useful for knowing how long we should keep the metal in the furnace to obtain the desired grain structure.

### 2.2. Multi-phase-field model for grain growth

We describe the dynamics of grain growth driven by the minimization of interfacial energy among grains by means of PF modeling [4–6]. The modeling characterizes the grain structure by a set of time-dependent field variables 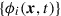, termed PF variables. 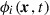 describes the probability of the existence of grain *i* at point 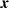 and time *t*, where the integer *i* specifies the type of grain to be distinguished. 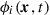 takes the value one when point 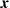 at time *t* is occupied only by grain *i* and zero when grain *i* does not exist. The case where 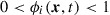 means that 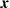 belongs to the grain boundary between grain *i* and other grains. Since 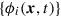 are existence probabilities, they must obey the normalization condition 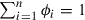, where *n* is the maximum number of different grains. This paper assumes that the PF variables obey the multi-phase-field (MPF) model proposed by Steinbach et al. [5]:(1)
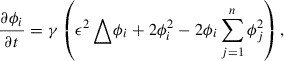



where 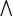 denotes a Laplacian operator, and 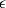 and 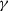 are the parameters to be determined. The parameter 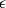 characterizes the thickness of the grain boundary, where the thickness is proportional to 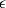. Considering that several snapshots of the grain structure can be obtained, 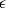 is a measurable parameter in this model. Thus, 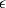 does not need to be estimated, and we use it as a unit of spacing. On the other hand, the parameter 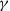 is an unobservable parameter in this model because we cannot estimate 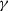 only by observing the snapshots. Thus, establishing a methodology to estimate 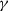 from limited amounts of observable data is needed, and has been an important task in the field of material science.

In order to compute the time evolution of 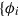 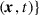, we discretize Equation (1) in rectangular two-dimensional (2D) space. Let 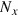 and 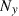 be the numbers of grid points along the *x*- and *y*-directions, respectively, 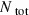 be the total number of grid points, i.e. 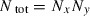, and *h* be the grid spacing. A periodic boundary condition is imposed on the boundary of the computational domain. Letting 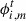 be the PF variable of grain *i* at the *m*th grid point and time *t*, Equation (1) can be rewritten as(2)
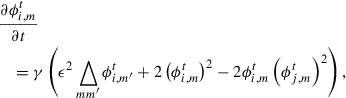



where the Einstein summation convention has been used for repeated subscripts not appearing on the left-hand side, and 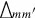 denotes a discrete Laplacian in the regular 2D grid.

**Figure 2.**
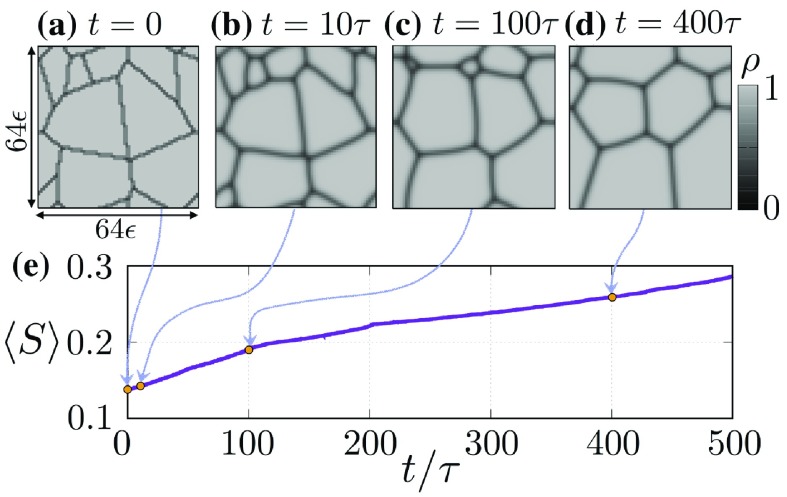
Temporal evolution of the grain structure and average grain size 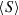 calculated by the MPF model with the initial condition shown in (a). Grayscale intensity in (a)–(d) indicates the magnitude of 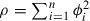.

Figures 2(a)–2(d) show the time evolution obtained by solving Equation (2) discretized in time by the Euler scheme with time step 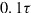, where 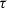 is the unit of time to be determined by an interval of observations. In this simulation, we used 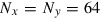, 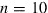, 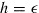, and 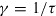. The initial state (Figure 2(a)) was set to be a Voronoi diagram generated from *n* randomly distributed parent points. The color in Figures 2(a)–2(d) indicates the magnitude of(3)
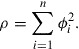



We find that the grain structure evolved to reduce the curvature of the grain boundaries, and larger grains grew to absorb smaller grains.

For the sake of measuring the grain structure quantitatively, we introduce the average grain size 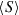 defined as(4)
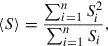



where we define the areal size 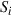 of grain *i* as(5)
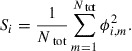



Figure 2(e) shows the time evolution of average grain size 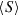, which grows monotonically in accordance with the growth of the grain structure, so that 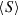 is useful for quantitatively evaluating growth.

## 3. Methodology

We propose a novel methodology for evaluating the grain structure at 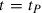 by using observational data based on Bayesian statistics. Since the methodology is based on the DA technique reported in [19], we explain how to apply the technique to the MPF model prior to explaining the methodology. The DA technique allows us to estimate simultaneously the grain structure and the parameter 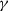 at an arbitrary time 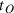, earlier than the time of the first observation 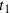. Furthermore, the technique, which is based on 4DVar, also allows us to obtain the uncertainty in the parameter 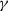. The 4DVar is composed of three procedures: (i) defining a ‘state-space model’ (SSM) and an ‘observation model’, (ii) constructing a probability density function (PDF) called a ‘posterior PDF’ based on Bayesian statistics, and (iii) optimization of the posterior PDF using an ‘adjoint model’. These procedures are explained in Sections 3.1–3.3. Section 3.4 describes the technique for quantifying the uncertainty in the parameter 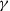. Finally, we explain our new methodology in Section 3.5.

### 3.1. The state-space model and the observation model

When implementing 4DVar, we must define an SSM, which is the time evolution of a large vector called the ‘state vector’ that contains variables involved in a given simulation model and, sometimes, the model parameters. The state vector used in this study is given by a time-dependent vector 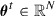 with elements(6)
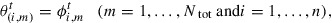



where we use the brief notation 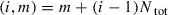 and(7)
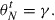

*N* is the total number of elements in the state vector, i.e. 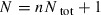. Equation (2) written in terms of 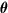 is given by(8)
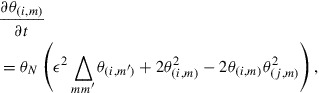



where we abbreviate the superscripts that describe time *t* for the sake of simplicity, except when the time must be explicitly specified. Moreover, we can write the time evolution of 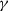 as(9)
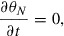



since the parameter 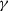 is not a time-dependent variable. This technique embeds the parameters involved in the simulation model into a large vector together with the time-dependent variables of the model, and allows us to estimate the parameters and the initial states of the variables simultaneously. The set of Equations (8) and (9) is the SSM considered in this study. Since the SSM indicates that the state vector 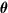 evolves autonomously, we are allowed to use a simple notation for the right-hand side, i.e.(10)
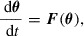



where 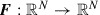 is a function of 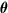. Note that the trajectory of 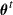 is determined only by controlling the initial state 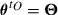, so that our purpose here is to search for the optimum 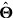 for 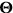 that best explains the time series of observational data.

In the construction of the methodology for DA, we need an observation model that describes the relation between observational data and theoretical values comparable with them. Supposing that we can detect the spatial distributions of PF variables from snapshots and that the data include noise, we write the relation between PF variables and observational data 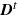 as(11)
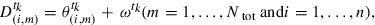



where 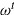 is the observation noise that occurs when keeping the metal in an isothermal environment. We assume that the observation noise is independent and identically distributed, and follows a normal distribution with mean zero and variance 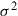.

### 3.2. Bayes’ theorem

In order to search for the optimum 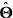, the 4DVar measures and maximizes the posterior PDF, which is the conditional PDF of 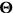 with a given 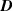. Bayes’ theorem states that the posterior PDF is given by(12)
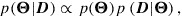



where 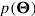 and 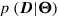 are called the prior PDF and the likelihood function, respectively.

The prior PDF describes prior information provided by experience and intuition. This paper assumes that prior information describes a constraint on 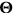 given by(13)
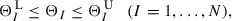



where 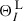 and 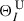 are the lower and upper bounds of 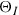, respectively. These bounds are given by experience, and some of them can be determined by conditions for the MPF model to be physically valid. Since PF variables indicate the existence probabilities of grains, we set the lower and upper bounds of these variables to 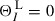 and 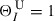 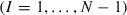, respectively. Furthermore, we set the lower bound 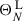 to zero because 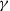 is a positive parameter. On the other hand, we cannot determine the upper bound 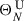 on the basis of conditions for the MPF model. In this paper, we set it as a large constant 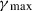. In computations to obtain estimates, it has been confirmed that the selection of 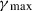 does not influence the estimation results. Assuming that the prior PDF 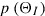 for 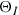 is uniform within the range given by Equation (13), we describe the joint prior PDF 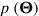 as(14)
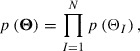



where(15)
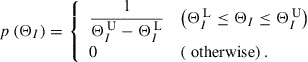



The likelihood function quantifies the difference between observational data and the corresponding theoretical values. The relation defined by Equation (11) allows us to describe the likelihood function as(16)
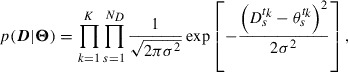



where 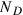 denotes the total number of data items, i.e. 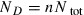, and the standard deviation 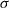 is interpreted as a hyper-parameter to be estimated.

Due to the convenience of numerical computation, we search for the optimum 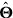 by minimizing cost function *J*:(17)
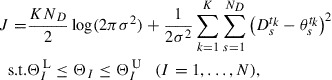



which is from a negative logarithm of 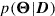, i.e. 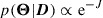, instead of maximizing 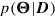. The constraint in Equation (17) arises from the negative logarithm of the prior PDF 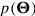, which appears when calculating 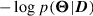. The optimum standard deviation 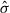 of 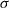 is determined by optimizing *J* with respect to 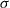. By solving 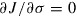, we obtain(18)
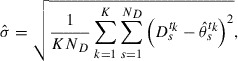



where 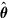 is the 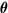 calculated by Equation (10) using 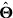. In practical computations, searching for 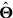 and 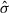 simultaneously is complex as 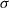 might make *J* singular. To avoid this difficulty, we decompose the optimization problem into two steps: (i) optimization of another cost function given by(19)
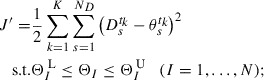



and (ii) the calculation of 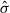 via Equation (18) after optimizing 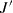. The optimization of the cost function 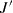 provides us the same solution as obtained by the optimization of *J*. For the definition of 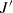, the posterior PDF into which the optimum 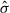 is substituted satisfies 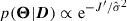.

### 3.3. Optimization utilizing an adjoint model

When searching for 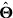 by optimizing the cost function 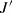 with the constraints, we should use gradient-based optimization, such as the L-BFGS-B method [29], because the number of dimensions of 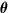 is large. However, the gradient 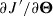 needed in gradient-based optimization cannot be calculated by directly differentiating 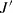 with respect to 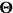, since 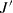 does not include 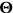 explicitly as seen in Equation (19). In 4DVar, we utilize an adjoint method to calculate the objective gradient. This method involves constructing the following three equations with respect to a time-dependent vector 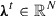:(20)
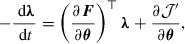

(21)
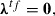

(22)
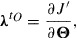



where 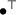 denotes the transpose of 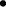, and 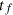 is an arbitrary time later than the time of the last observation 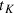. 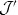 is a time-dependent function satisfying(23)
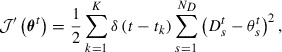



where 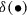 stands for the Dirac delta function. The details of the derivation of these equations can be found in [24–26]. The set of Equations (20)–(22), which is called the adjoint model, states that we can obtain the objective gradient 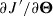 as the initial condition of 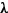 (Equation 22) by solving Equation (20) backward in time, with the final condition given by Equation (21). By utilizing the gradient obtained by solving the adjoint model in the iteration of gradient-based optimization, we can search for 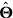 quickly even when the dimensionality of 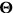 is large. The number of computations needed to solve the adjoint model is proportional to the same order of the computations needed to solve the simulation model (Equation 10), which is denoted by *C*. Thus, searching for the optimum 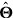 can be dealt with by a number of computations proportional to 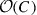. In Appendix A.1, we present a tangible description of the adjoint model where 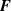 is not used.

### 3.4. Uncertainty quantification utilizing a second-order adjoint method

The conventional 4DVar described in Section 3.1 allows us to estimate the optimum 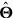 quickly even when the dimensionality of 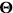 is large. However, it does not provide us information about the uncertainty in 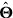. To extract this information, another procedure must be implemented on the conventional 4DVar. Ito et al. [19] recently developed a methodology to solve this problem by combining Laplace’s method and the SOA method [26–28] that efficiently evaluates the second derivative of the cost function. The methodology allows us to extract an uncertainty of interest more efficiently than conventional approaches, and hence is appropriate for our purposes, i.e. evaluating the uncertainty in the parameter 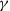.

This section briefly explains the essence of the methodology. Having obtained the optimum 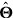 by optimization using the conventional 4DVar, we assess the posterior PDF 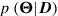 in the neighborhood of 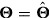. Given that gradient 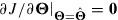, an approximation of 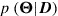 can be obtained by Laplace’s method as a multivariate normal distribution:(24)
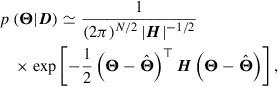



where 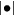 denotes the determinant of 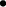, and 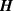 is a Hessian matrix of *J* given by(25)
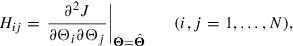



or, alternatively,(26)
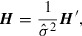



for the definition of 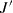. 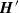 is a Hessian matrix of 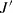, i.e.(27)
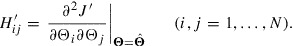



Let *l* 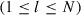 be an index of the component of interest in 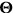. Integrating Equation (23 24) over all variables except 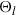, we obtain a marginal PDF with respect to 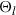 as(28)
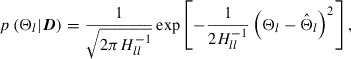



where 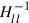 is the *l*th diagonal element in the inverse of the Hessian matrix 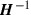. This equation means that the variance with respect to 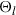 can be obtained by extracting 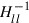 from 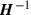. However, when *N* is massive, it is unrealistic to calculate 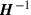 directly, as this requires 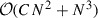 computations and 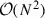 memory. To save on the computational cost of obtaining 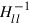, the methodology considers the following linear equation of 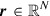:(29)
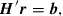



where 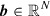 is a vector with elements 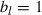 and 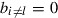. The solution 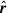 includes 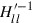 as 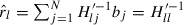, and the objective standard deviation 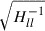 is calculated by 
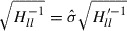
. The linear equation shown in Equation (29) can be solved by an iterative technique utilizing the Krylov subspace, such as the conjugate gradient method or the conjugate residual method. The iterative method utilizing the Krylov subspace needs to compute many Hessian-vector products. The SOA method can compute these Hessian-vector products avoiding the computations of all elements of the matrix. The SOA method considers the time evolutions of two time-dependent vectors 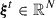 and 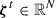, given by(30)
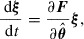



and(31)
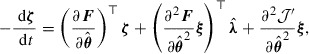



where 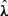 corresponds to 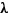 obtained by solving Equation (20) with 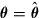, and the 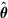-derivatives represent the 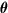-derivatives into which 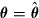 is substituted. A combination of Equations (30) and (31), which are called the ‘tangent linear model’ (TL) and the ‘SOA model’, respectively, allows us to compute the Hessian-vector product for an arbitrary vector. Solving Equation (30) in the forward direction of time with a given vector 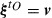, we obtain the time series of 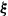. Then, solving Equation (31) backward in time with 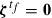 and the time series of 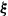, we obtain the objective Hessian-vector product as 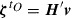. See [27] for the detailed derivation of Equations (30) and (31). The number of computations used to solve these models is proportional to 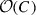, so that obtaining an uncertainty in 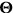 by solving Equation (29) with an iterative method is proportional to 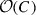.

This paper aims to obtain the uncertainty 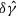 in the estimated parameter 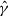, and hence we associate *l* with the index of 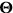 that corresponds to 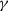, i.e. 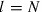. The procedure used to obtain the uncertainty 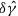 can be summarized as follows.

Step 1. Prepare a vector 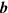 with elements 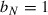 and 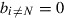.
Step 2. Solve Equation (29) via an iterative method utilizing a Krylov subspace. The Hessian-vector products needed in the Krylov subspace method are computed by solving Equations (30) and (31).
Step 3. Extract 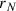 from the solution 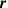 of Equation (29) and compute the uncertainty by 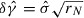, where 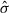 can be computed by Equation (18).

Appendix A.2 presents the derivations of the TL and the SOA models for the MPF model.

### 3.5. Prediction of grain structure

The 4DVar-based methodology presented in Sections 3.1–3.4 enables us to determine the optimum estimates that maximize the posterior PDF together with their uncertainties. This section presents how the predictive problem is solved through the obtained optimum estimates and the uncertainties.

Let 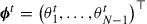 be a vector describing the grain structure at time *t*. In the prediction methodology, we first evaluate the optimum 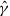 for 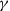 and its uncertainty 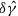 by the 4DVar-based methodology, where the initial time 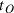 is set to 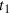. Since we know the initial grain structure accurately in advance of placing the sample in the heating furnace, we utilize the grain structure as the ‘true’ structure 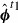, i.e. we fix 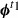 to 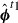 in the optimization of the 4DVar-based methodology.

The simplest way to obtain the predicted grain structure 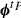 is to solve the MPF model until 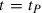 with the optimum parameter 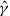, starting from 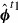. However, it is not adequate from the perspective of the evaluation of 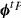 because the error in the parameter influences the forecast of 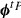. Thus, we calculate the change in the forecast of 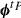 when using a value of 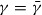 slightly deviating from 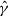, by associating the change with the time evolution 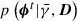, which is a conditional PDF of 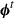 for a given 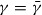 and observational data 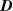. Although the time evolution of the conditional PDF, in principle, can be computed by using a sampling method, such as a particle filter, it is unrealistic as the number of dimensions of 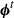 is large. Thus, we employ a strategy whereby we track only the evolution of the maximizer of 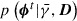, which is the solution that maximizes the PDF, instead of directly calculating 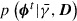. This strategy can help save a large number of computations, as the complexity of obtaining the time evolution of the maximizer is 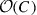, as described below.

The strategy requires the construction of the time evolution equation of the maximizer from that of 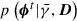. To evolve 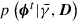, we first need to define the initial PDF 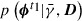. It is linked to the initial joint PDF 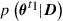 and described as(32)
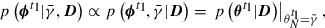



where the initial joint PDF is equivalent to 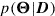 shown in Equation (23 24). However, employing the right-hand side of Equation (32) as the initial PDF makes the computation difficult as we must completely calculate the time evolution of 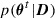. Thus, we consider approximating 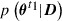 by a multivariate normal distribution 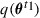 with mean vector 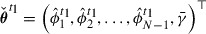 and covariance matrix 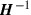. Since 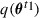 satisfactorily approximates 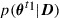 when 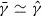, we consider the time evolution of 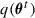, rather than that of 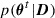, by an integral form using a small time step 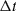 as(33)
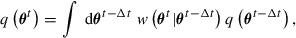



where the kernel function 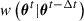 provides a mapping from 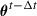 to 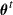. Since the mapping is given by the discretized Equation (10),(34)
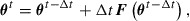



the kernel function becomes(35)
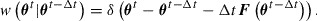



The combination of Equations (33) and (35) provides a transformation from 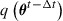 to 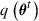, meaning that 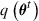 for any *t* can be calculated in principle by recursively using Equations (33) and (35), starting at the initial PDF. However, the direct computation of 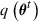 is difficult due to the large number of dimensions of 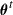 and the nonlinearity of 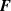. Thus, we extract the time evolution equation of the maximizer from that of 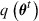 by linearizing Equation (34). Let 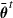 be the maximizer of 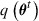, and 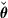 be the state vector obtained by solving Equation (34) with given 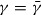 and initial state 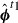. A Taylor expansion of Equation (34) in the neighborhood of 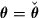 is given by(36)
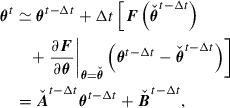



where both matrices 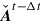 and 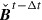 are functions of 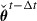. The use of Equation (36) instead of Equation (34) provides an approximation of the kernel function as(37)
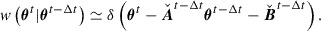



The kernel function that describes a linear mapping ensures that when 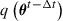 is a multivariate normal distribution, 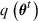 is another multivariate normal distribution. Since 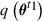 is a multivariate normal distribution, the time evolution equation of the maximizer 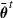 is obtained by substituting Equation (37) by Equation (33) and then calculating its expectation,(38)
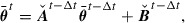



where the initial condition is given by 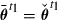. Note that we need to solve Equations (34) and (38) simultaneously to obtain the time evolution of 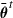. The computational complexity is 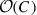 in this case, so that we can save a large number of computations compared with the direct computation of the time evolution of 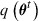, the complexity of which is proportional to 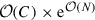.

In the next step, we associate 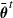 with the maximizer of 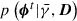. Let 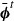 be the maximizer of 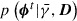. Considering that 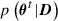 is approximated by 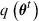, 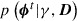 is described as(39)
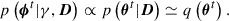



The linear mapping ensures that 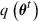 is a multivariate normal distribution(40)
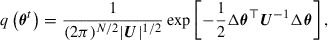



where 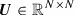 is a covariance matrix, and 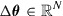 is a residual vector with elements 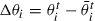 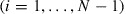 and 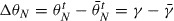. Substituting Equation (40) and 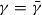 by Equation (39) and subsequently calculating its expectation, 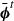 becomes consistent with the elements of 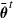, i.e. 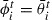 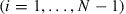. Note that the calculation of the expectation does not require the covariance matrix 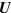. In summary, the procedure to obtain the maximizer of 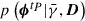 consists of the following three steps.

Step 1. Set 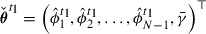 and 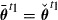.
Step 2. Evolve 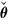 and 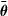 simultaneously from 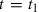 to 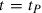 by using Equation (34) for 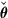 and Equation (38) for 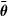.
Step 3. Extract 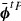 from 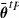.

The use of this procedure together with the method in Section 3.4 enables us to evaluate the uncertainty in prediction from parameter uncertainties. This paper evaluates 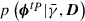 by giving 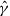 and 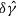, i.e. it calculates three vectors 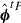, 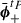, and 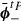, which are the maximizers of 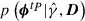, 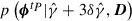, and 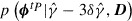, respectively.

## 4. Setup for estimation tests

We apply the proposed methodology to the MPF model to assess its performance by using estimation tests involving a time series of synthetic data. The synthetic data are created by the following procedures: (i) we prepare a time series of 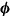 with a given initial state 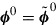 and parameter 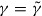 (we call this time series the ‘true trajectory’); (ii) we extract 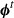 at time 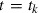 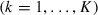 from the calculated time series; and (iii) we then add noise that follows a normal distribution with mean zero and variance 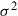. Our methodology is validated if it can reproduce 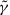 and accurately estimate 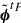. We call this type of estimation test a ‘twin experiment’. Throughout the experiments reported in this paper, we use the grain structure shown in Figure 2(a) as 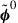 and 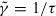. When performing the twin experiments, we need to calculate the temporal evolutions of the adjoint model, the TL model, and the SOA model, by discretizing them. For simplicity, we discretize them in time with the Euler scheme, and use the same time step and space resolution as the ones used in Section 2.2. To ensure that each element in 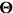 satisfies the constraint (Equation 13), 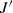 is optimized by using the L-BFGS-B technique [29]. The upper bound 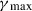 of the constraint on 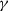 described in Section 3.2 is set to 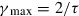. When we quantify the uncertainty in 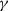, we use the conjugate residual method as the Krylov subspace method.

## 5. Results and discussion

The proposed method is validated through two types of twin experiments: (I) estimation of parameter 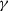 conditional on fixing 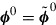, and (II) evaluation of 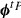. Test I determines whether the 4DVar-based methodology works well by investigating how the estimations depend on observational data. Test II investigates and quantifies how the prediction of the grain structure depends on the estimations.

### 5.1. Test I: Parameter estimation

Test I investigates the influences of three parameters related to the observational data: (i) the interval of observation 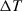; (ii) the standard deviation of observation noise 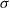; and (iii) the length of observation time 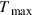. In this test, we assume that the observations are obtained at equal intervals 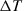, i.e. the number of observations *K* is given by 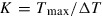, and the observation times are given by 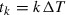 (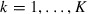). The observational parameters used in Test I are shown in Table 1.

**Table 1.** Values of the interval of observation 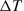, the standard deviation of observation noise 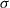, and the length of observation time 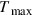 used in Test I. Tests I(i), I(ii), and I(iii) investigated how the estimation depended on 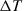, 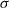, and 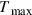.

	Test I(i)	Test I(ii)	Test I(iii)
	–		
		–	
			–

**Figure 3.**
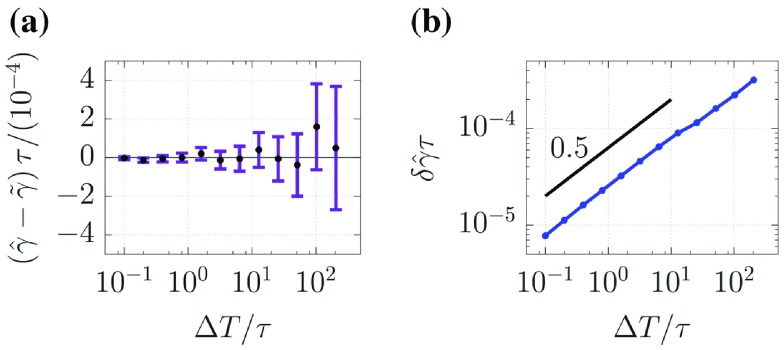
Results of Test I(i). The length of each error bar for optimum parameter 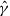 in (a) corresponds to the estimated uncertainty 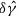 in (b). The black solid line in (b) indicates a square root function.

**Figure 4.**
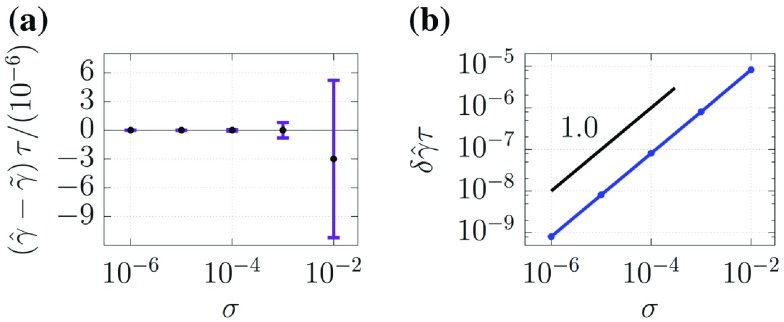
Results of Test I(ii). The length of each error bar for optimum parameter 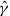 in (a) corresponds to the estimated uncertainty 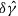 in (b). The black solid line in (b) indicates a linear function.

**Figure 5.**
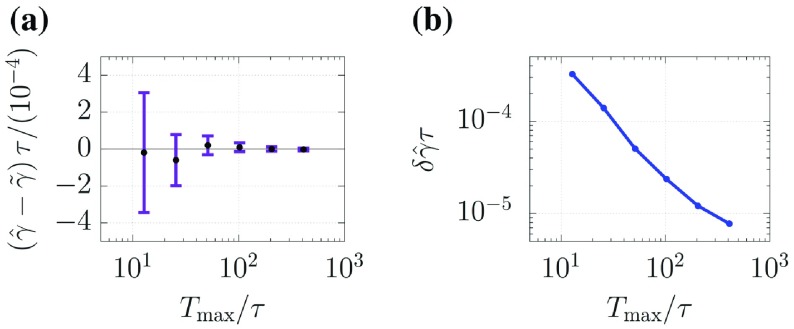
Results of Test I(iii). The length of each error bar for optimum parameter 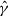 in (a) corresponds to the estimated uncertainty 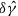 in (b).

**Table 2.** Values of the interval of observation 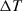 used in Test II.

	Test II(i)	Test II(ii)	Test II(iii)
			

**Figure 6.**
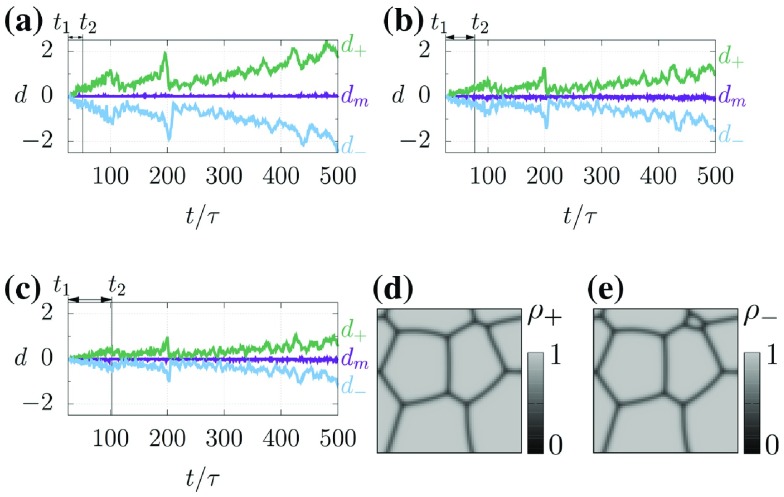
Results of Test II. (a)–(c) indicate the predictions of the normalized variations *d*. (d) and (e) correspond to the predicted grain structures 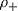 and 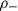 at 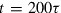 in Test II(i), respectively.

Figure 3 shows the results of Test I(i). Figure 3(a) indicates that parameter estimation is successful as the estimated parameter is in the range 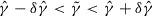. Figure 3(b) indicates that 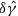 is a monotonically increasing function of 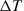, and is proportional to 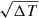 for 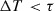.

Figure 4 shows the results of Test I(ii). Figure 4(a) indicates that the 4DVar-based method is also successful in parameter estimation, and Figure 3(b) shows that 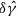 is proportional to 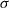. The power laws observed in Figures 3(b) and 4(b) become fine in accordance with increasing number of observations (i.e. 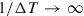) or decreasing roughness of data (i.e. 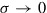).

Figure 5 shows the results of Test I(iii). Figure 5(a) indicates that parameter estimation is satisfactory. Figure 5(b) shows that 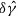 is a monotonically decreasing function of 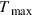 that does not obey any power laws in the range, and its functional form appears to saturate to a constant when 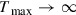.

These features of the uncertainty 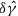 can be explained by the following theoretical consideration of the Hessian matrix. Having obtained the optimum 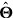 by optimization using the conventional 4DVar described in Section 3.3, the Hessian matrix at the optimum 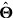 can be described by a formal solution given by(41)
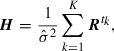



where 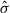 is obtained by Equation (18). The time-dependent matrix 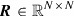 is given by(42)
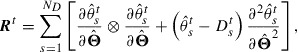



where 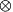 denotes a direct product. Letting the number of observations be infinity with a fixed length of observation time, i.e. 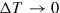 and 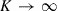 such that 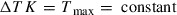, the limitation of the Hessian matrix becomes(43)
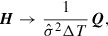



where a matrix 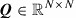 is given by(44)
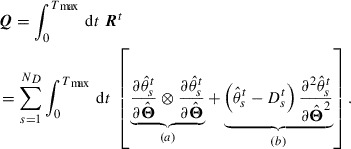



As 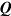 does not depend on 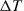 or 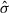, we immediately find, from the functional form of Equation (43), that the uncertainty is proportional to 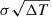 on the assumption that 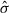 converges with 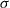 when 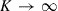. This fact qualitatively explains the power laws associated with 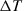 and 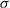 shown in Figures 3(b) and 4(b), respectively. Furthermore, as 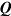 includes all information pertaining to the simulation model, the power laws are naturally independent of the details of the model. The 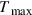-dependency of the uncertainty can be described by 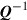. Although it is difficult to evaluate 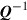 analytically, its long-term behavior can be discussed. As seen in Equation (44), the diagonal elements in term (a) are always positive, whereas the off-diagonal elements in term (a) and all elements in term (b) can be either positive or negative. When 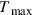 is significantly large, the diagonal elements of 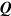 grow to positive values larger than the off-diagonal elements, and this growth continues until 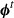 is uniform in the computational domain. That is, 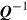 approaches a constant diagonal matrix when 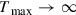, and this causes the saturation of the uncertainty. For this reason, we consider that the uncertainty shown in Figure 5(b) approaches a constant value when 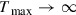.

### 5.2. Test II: Prediction of grain structure.

In Test II, we use three types of synthetic datasets parameterized by interval 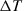. The synthetic dataset is assumed to be composed of two snapshots of grain structures (i.e. 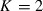) at times 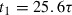 and 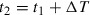, where the list of 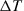 used in this test is summarized in Table 2. The standard deviation 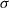 for generating the synthetic data is set to 0.1. Using the synthetic dataset, we estimate the parameter 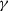 together with its uncertainty, and then apply our prediction method. In the three tests, we confirmed that the uncertainties were significantly small compared with the estimated parameters.

Figure 6 shows the results of Test II. To quantify the differences between Tests II(i)–II(iii), we investigate the temporal evolution of the normalized variation of the average grain size given by(45)
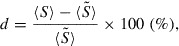



where 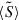 indicates the average grain size calculated by using the PF variables that obeyed the true trajectory. Let 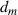, 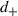, and 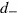 be the normalized variations using the PF variables 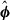, 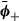, and 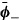, respectively. Figures 6(a)–6(c) indicate the trajectories of the normalized variations in Tests II(i)–II(iii). The estimation of the grain structure is successful because the trajectory of 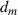 is almost zero. Furthermore, we find that the trajectories of 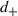 and 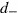 enclose that of 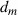, meaning that our methodology is successful in providing the upper/lower realizations of the grain structure. The deviations in 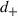 and 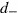 from 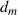 tend to grow over time, and their growth rates are smaller in accordance with the magnitude of 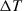. These results imply that using a larger 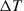 provides better estimation of grain structure because 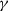 characterizes the speed of the grain boundary, meaning that using snapshots of structures that are significantly different is advantageous for estimating 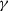. Furthermore, 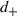 and 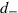 fluctuate in their trajectories, indicating that the normalized variation is sensitive to that in the average grain size. In particular, the large reductions of the deviations appear at 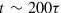 in Figures 6(a)–6(c). These phenomena can be explained by considering the dynamical properties of the MPF model. Figures 6(d) and 6(e) show the predicted grain structures 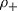 and 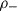, respectively, at 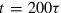 in Test II(i), which are 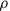 calculated by 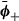 and 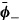, respectively. They show a small grain in 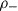 that is not observed in 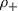. In general, once small grains have disappeared, the grain boundaries around multiple points rapidly vary to their equilibrium forms (i.e. triplet points with a 120-degree structure). Once the 120-degree structure has been formed, the growth/decay of grains slows down. This implies that the grain structures 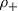 and 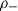, once the small grains have disappeared, tend to be synchronized with each other. The large reduction in the deviation at 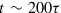 is due to this synchronization.

## 6. Conclusions

This paper proposed a 4DVar-based DA procedure applicable to the MPF model that describes purely interface-driven grain growth. We adopted the 4DVar technique proposed by Ito et al. [19] that allows us to obtain the optimum estimates together with their uncertainties within realistic computational time and resources, even in the case of massive simulation models like the MPF model. The procedure properly estimated parameters of the MPF model together with their uncertainties. Moreover, we devised a method to determine how a perturbation of the parameter influences the prediction of the temporal evolution of grain structure. The method allows us to quantify the uncertainty propagation of the parameter by combining the 4DVar technique proposed by Ito et al. We tested the method through numerical tests and quantified how the observational conditions influenced the prediction of the temporal evolution of grain structure. These results are expected to provide valuable information related to the mechanical properties of metals.

Further investigation into the uncertainty in the prediction can improve the design of the observational setup. For example, we can optimize a distribution of sensors and/or observation frequencies that minimizes prediction uncertainties. Such an investigation can allow us to attain a cost-effective development of materials within limited observational time and resources. Furthermore, the proposed prediction method is applicable not only to the interface-driven MPF model, but also to other PF models that involve more complex physics, such as various types of chemical reactions, and thermal and elastic conditions. Through such applications, we can predict the future states of the key factors related to mechanical properties, such as the spatial distributions of chemical compositions and the residual stress in the metal, as well as the grain structure. The 4DVar technique can also estimate past states by shifting the time of origin 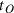 to before the first observational time 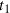. This enables us to uncover the histories of the mechanical properties and then pursue the causality that explains the given state of the metal. We believe that such applications will contribute to the efficient development of materials in the future.

## Appendix 1

### Models for data assimilation

This appendix presents the tangible descriptions of the adjoint model, the TL model, and the SOA model used for the construction of our methodology in the main body of the paper.

#### Derivation of the adjoint model

Substituting the right-hand sides shown in Equations (8) and (9) for  as shown in Equation (20), the adjoint model can be given as

(A1)

(A2)

(A3)

#### Derivation of the tangent linear model and the second-order adjoint model

The TL model and the SOA model are derived in the same manner as the adjoint model shown in Appendix A.1. The TL model is given by

(A4)

and the SOA model by

(A5)

## References

[CIT0001] Manohar PA, Dunne DP, Chandra T, et al Grain growth predictions in microalloyed steels. ISIJ Int. 1996;36(2):194–200. DOI:10.2355/isijinternational.36.194.

[CIT0002] Uhm S, Moon J, Lee C, et al Prediction model for the austenite grain size in the coarse grained heat affected zone of Fe-C-Mn steels: considering the effect of initial grain size on isothermal growth behavior. ISIJ Int. 2004;44(7):1230–1237. DOI:10.2355/isijinternational.44.1230.

[CIT0003] Lee SJ, Lee YK Prediction of austenite grain growth during austenitization of low alloy steels. Mater Design. 2008;29(9):1840–1844. DOI:10.1016/j.matdes.2008.03.009.

[CIT0004] Kobayashi R Modeling and numerical simulations of dendritic crystal growth. Physica D. 1993;63(3):410–423. DOI:10.1016/0167-2789(93)90120-P.

[CIT0005] Steinbach I, Pezzolla F, Nestler B, et al A phase field concept for multiphase systems. Physica D. 1996;94(3):135–147. DOI:10.1016/0167-2789(95)00298-7.

[CIT0006] Steinbach I, Pezzolla F A generalized field method for multiphase transformations using interface fields. Physica D. 1999;134(4):385–393. DOI:10.1016/S0167-2789(99)00129-3.

[CIT0007] Kim SG, Kim DI, Kim WT, et al Computer simulations of two-dimensional and three-dimensional ideal grain growth. Phys Rev E. 2006 Dec;74:061605 DOI:10.1103/PhysRevE.74.061605. 17280076

[CIT0008] Boettinger WJ, Warren JA, Beckermann C, et al Phase-field simulation of solidification. Ann Rev Mater Res. 2002;32(1):163–194. DOI:10.1146/annurev.matsci.32.101901.155803.

[CIT0009] Chen LQ Phase-field models for microstructure evolution. Ann Rev Mater Res. 2002;32(1):113–140. DOI:10.1146/annurev.matsci.32.112001.132041.

[CIT0010] Ohno M, Yamaguchi T, Sato D, et al Existence or nonexistence of thermal pinning effect in grain growth under temperature gradient. Comput Mater Sci. 2013;69:7–13. DOI:10.1016/j.commatsci.2012.11.017.

[CIT0011] Reich S, Cotter C Probabilistic forecasting and bayesian data assimilation. Cambridge: Cambridge University Press; 2015.

[CIT0012] Ghil M, Malanotte-Rizzoli P Data assimilation in meteorology and oceanography. Adv Geophys. 1991;33:141–266. DOI:10.1016/S0065-2687(08)60442-2.

[CIT0013] Kalnay E Atmospheric modeling, data assimilation and predictability. Cambridge: Cambridge University Press; 2003.

[CIT0014] Tsuyuki T, Miyoshi T Recent progress of data assimilation methods in meteorology. J Meteorol Soc Jpn Ser. 2007;II(85B):331–361. DOI:10.2151/jmsj.85B.331.

[CIT0015] Ueno G, Higuchi T, Kagimoto T, et al Application of the ensemble Kalman filter and smoother to a coupled atmosphere-ocean model. SOLA. 2007;3:5–8. DOI:10.2151/sola.2007-002.

[CIT0016] Nagao H, Higuchi T, Miura S, et al Time-series modeling of tide gauge records for monitoring of the crustal activities related to oceanic trench earthquakes around japan. Comput J. 2013;56(3):355–364. DOI:10.1093/comjnl/bxs139.

[CIT0017] Kano M, Nagao H, Ishikawa D, et al Seismic wavefield imaging based on the replica exchange Monte Carlo method. Geophys J Int. 2017;208(1):529 DOI:10.1093/gji/ggw410.

[CIT0018] Maeda T, Obara K, Shinohara M, et al Successive estimation of a tsunami wavefield without earthquake source data: a data assimilation approach toward real-time tsunami forecasting. Geophys Res Lett. 2015;42(19):7923–7932. DOI:10.1002/2015GL065588.

[CIT0019] Ito S, Nagao H, Yamanaka A, et al Data assimilation for massive autonomous systems based on a second-order adjoint method. Phys Rev E. 2016 Oct;94:043307 DOI:10.1103/PhysRevE.94.043307. 27841577

[CIT0020] Doucet A, Godsill S, Andrieu C On sequential Monte Carlo sampling methods for Bayesian filtering. Stat Comput. 2000;10(3):197–208. DOI:10.1023/A:1008935410038.

[CIT0021] Kitagawa G Introduction to time series modeling. Chapman & Hall/CRC Monographs on Statistics & Applied Probability, Boca Raton, FL: CRC Press; 2010.

[CIT0022] Houtekamer PL, Mitchell HL Data assimilation using an ensemble Kalman filter technique. Mon Weather Rev. 1998;126(3):796–811. DOI:10.1175/1520-0493(1998)126\lt0796:DAUAEK\gt2.0.CO;2.

[CIT0023] Evensen G The ensemble Kalman filter: theoretical formulation and practical implementation. Ocean Dyn. 2003;53(4):343–367. DOI:10.1007/s10236-003-0036-9.

[CIT0024] Le Dimet FX, Talagrand O Variational algorithms for analysis and assimilation of meteorological observations: theoretical aspects. Tellus A. 1986;38A(2):97–110. DOI:10.1111/j.1600-0870.1986.tb00459.x.

[CIT0025] Talagrand O, Courtier P Variational assimilation of meteorological observations with the adjoint vorticity equation. I: theory.Q J R Meteorol Soc. 1987;113(478):1311–1328. DOI:10.1002/qj.49711347812.

[CIT0026] Wang Z, Navon I, Le Dimet F, et al The second order adjoint analysis: theory and applications. Meteorol Atmos Phys. 1992;50(1–3):3–20. DOI:10.1007/BF01025501.

[CIT0027] Wang Z, Droegemeier K, White L The adjoint Newton algorithm for large-scale unconstrained optimization in meteorology applications. Comput Optim Appl. 1998;10(3):283–320. DOI:10.1023/A:1018321307393.

[CIT0028] Le Dimet FX, Navon I, Daescu DN Second-order information in data assimilation*. Mon Weather Rev. 2002;130(3):629–648. DOI:10.1175/1520-0493(2002)130\lt0629:SOIIDA\gt2.0.CO;2.

[CIT0029] Byrd RH, Lu P, Nocedal J, et al A limited memory algorithm for bound constrained optimization. SIAM J Sci Comput. 1995;16(5):1190–1208. DOI:10.1137/0916069.

